# Efficacy of a liquid low-energy formula diet in achieving preoperative target weight loss before bariatric surgery

**DOI:** 10.1017/jns.2016.13

**Published:** 2016-05-30

**Authors:** Lone V. Nielsen, Mette S. Nielsen, Julie B. Schmidt, Sue D. Pedersen, Anders Sjödin

**Affiliations:** 1 Department of Nutrition, Exercise and Sports, Faculty of Science, University of Copenhagen, Denmark; 2 C-ENDO Endocrinology Clinic, Calgary, AB, Canada

**Keywords:** Low-energy diets, Liquid formula diets, Preoperative weight loss, Bariatric surgery, BS, bariatric surgery, DXA, dual-energy X-ray absorptiometry, E%, energy percentage, FFM, fat-free mass, HIBW, higher initial body weight, LCD, low-energy formula diet, LIBW, lower initial body weight

## Abstract

A preoperative weight loss of 8 % is a prerequisite to undergo bariatric surgery (BS) in Denmark. The aim of the present study was to evaluate the efficacy of a 7- or an 11-week low-energy diet (LCD) for achieving preoperative target weight before BS. A total of thirty obese patients (BMI 46·0 (sd 4·4) kg/m^2^) followed an LCD (Cambridge Weight Plan^®^, 4184 kJ/d (1000 kcal/d)) for 7 or 11 weeks as preparation for BS. Anthropometric measurements including body composition (dual-energy X-ray absorptiometry), blood parameters and blood pressure were assessed at weeks 0, 7 and 11. At week 7, the majority of patients (77 %) had reached their target weight, and this was achieved after 5·4 (sem 0·3) weeks. Mean weight loss was 9·3 (sem 0·5) % (*P* < 0·01) and consisted of 41·6 % fat-free mass (FFM) and 58·4 % fat mass. The weight loss was accompanied by a decrease in systolic and diastolic blood pressure (7·1 (sem 2·3) and 7·3 (sem 1·8) mmHg, respectively, all *P* < 0·01) as well as an improved metabolic profile (8·2 (sem 1·8) % decrease in fasting glucose (*P* < 0·01), 28·6 (sem 6·4) % decrease in fasting insulin (*P* < 0·01), 23·1 (sem 2·2) % decrease in LDL (*P* < 0·01), and 9·7 (sem 4·7) % decrease in TAG (*P* < 0·05)). Weight, FFM and fat mass continued to decrease from week 7 to 11 (all *P* < 0·01), whereas no additional improvements was observed in the metabolic parameters. Severely obese patients can safely achieve preoperative target weight on an LCD within 7 weeks as part of preparation for BS. However, the considerable reduction in FFM in severely obese subjects needs further investigation.

The only long-term effective treatment currently proven to overcome severe obesity is bariatric surgery (BS)^(^^1^^)^. Studies have shown that the complexity of the surgical procedure and the risk of perioperative and postoperative complications are associated with the degree of obesity^(^^2^^,^^3^^)^.

Benefits of preoperative weight loss include a reduction in the risk of perioperative complications by reducing visceral adipose tissue and intrahepatic fat content, thereby minimising the risk of having to convert to open surgery^(^^2^^,^^4^^–^^6^^)^, shortening of the operating time^(^^7^^–^^10^^)^, as well as duration of hospitalisation^(^^10^^,^^11^^)^. In addition, a preoperative weight loss may even be associated with improved postoperative weight loss^(^^7^^,^^10^^,^^11^^)^, potentially by reflecting patients’ motivation, but also by accustoming patients to a lifestyle of food restriction after surgery. Therefore, in order to optimise the safety and efficacy of BS in severely obese patients, preoperative weight loss is preferable.

Most surgeons advise patients to lose weight prior to BS. The Danish Ministry of Health requires an 8 % weight loss for patients scheduled for BS^(^^12^^)^; however, there is currently no standard diet programme prescribed to obtain preoperative target weight. A weight loss achieved with conventional diet and lifestyle changes can be very difficult for some patients to obtain, especially in a pre-specified time. In such cases, a low-energy formula diet (LCD) with high protein content may be an effective option. These formulas typically limit energy intake to 3347–6276 kJ/d (800–1500 kcal/d), but contain all essential nutrients. Low-energy, high-protein formulas are able to induce rapid weight loss, often provide relatively adequate satiety after a few days, and attempt to minimise loss of fat-free mass (FFM)^(^^13^^,^^14^^)^. As more than 300 000 BS procedures are performed annually worldwide^(^^15^^)^, this intensifies the necessity for research with focus on improving preoperative weight-loss strategies.

In this study we aimed to determine the efficacy of an LCD to obtain an 8 % weight loss in severely obese patients prior to BS. Secondarily, potential health benefits and side effects of the LCD were evaluated.

## Methods

This study was a part of a larger study; a detailed description of the methods has recently been published elsewhere^(^^16^^)^.

### Study population

A total of thirty Caucasians, aged 18–65 years, with BMI ≥ 40 or BMI ≥ 35 kg/m^2^ combined with obstructive sleep apnoea or hypertension approved for Roux-en-Y gastric bypass were recruited from November 2009 to August 2011. Exclusion criteria were either related to endpoints in the main study and included diabetes mellitus, thyroid dysfunction, hypothalamic or genetic aetiology of obesity^(^^16^^)^, or contraindications related to the use of the LCD: previous case of diverticulitis, past history of ventricular arrhythmias, renal dysfunction (creatinine clearance <60 ml/min), elevated liver enzymes (alanine transaminase and aspartate transaminase >3× upper normal limit), milk protein allergy or lactose intolerance, porphyria or phenylketonuria, history of gout, breast feeding, concomitant use of monoamine oxidase inhibitors or non-potassium-sparing diuretics, or inability or unwillingness to comply with the LCD.

This study was conducted according to the guidelines laid down in the Declaration of Helsinki and all procedures involving human subjects were approved by the Municipal Ethical Committee of Copenhagen/Scientific Ethics Committee of the Metropolitan regions of Denmark (journal no. H-2-2009-091), the Danish Data Protection Agency (journal no. 2007-54-0296) and was registered at clinicaltrials.gov (ClinicalTrials.gov ID NCT00939679). Written informed consent was obtained from all of the subjects.

**Fig. 1.**
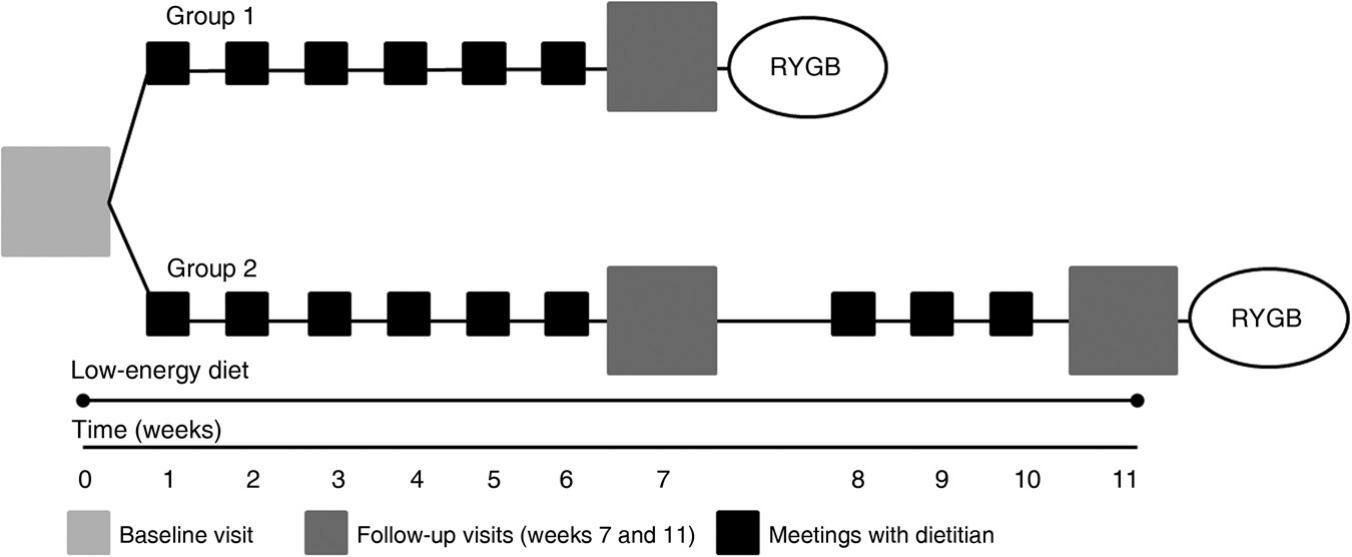
Overview of the study design. Since the design of the main study was a controlled trial, the participants were randomised to Roux-en-Y gastric bypass (RYGB) surgery in week 8 (group 1) or week 12 (group 2). Participants followed a low-energy formula diet from week 0 to week 11.

The study included a baseline visit (week 0) and two follow-up visits at weeks 7 and 11. In order to investigate effects on the primary endpoint of the main study (energy expenditure)^(^^16^^)^, subjects were randomised to one of two groups: group 1 scheduled for Roux-en-Y gastric bypass surgery at week 8 (*n* 15), or group 2 scheduled for Roux-en-Y gastric bypass surgery at week 12 (*n* 15). All participants received an LCD for 11 weeks. In the present paper only the pre-surgical periods will be examined: i.e. the period from week 0 to 7 (including both groups 1 and 2, *n* 30) and the period from week 7 to 11 (group 2 only, *n* 15) (Fig. 1). Subjects received weekly nutritional counselling from a dietitian.

### Screening

Patients eligible for the study were identified by endocrinologists at Hvidovre Hospital, Copenhagen, Denmark and referred to a screening visit at the Department of Nutrition, Exercise and Sports, University of Copenhagen, where protocol details were explained and a baseline visit was scheduled for eligible subjects.

### Baseline (week 0) and follow-up visits (weeks 7 and 11)

At the baseline visit demographic data and medical history were obtained, and patients were randomised to surgery at either week 8 or 12. Anthropometric data were collected, a fasting blood sample was drawn, and heart rate and blood pressure were measured with subjects rested and in the supine position. The LCD was initiated with their first visit with the dietitian.

The baseline visit and the two follow-up visits were identical.

### Low-energy diet

The LCD consisted of four powder-based meals (Cambridge Weight Plan^®^), 1 litre skimmed milk, 295 g vegetables and 100 g low-fat yoghurt per d, providing 4310 kJ/d (1030 kcal/d) (48 % energy (E%) from carbohydrates, 39 E% from protein, 13 E% from fat, and ≥100 % recommended daily allowances of all vitamins and minerals). Self-evaluated side effects and compliance with the LCD was recorded at the weekly meeting with the dietitian. Compliance was rated on a scale from 1 to 5, with 5 indicating ‘very good compliance’.

### Anthropometric measurements

Subjects were informed to wear lightweight clothing, no shoes, have an emptied bladder and to be fasted for their anthropometric assessment. Body weight was measured to the nearest 0·1 kg. Waist and hip circumferences were measured twice to the nearest 0·5 cm and the average was used. Height was measured to the nearest 0·5 cm using a wall-mounted digital stadiometer. Body composition was determined by dual-energy X-ray absorptiometry (DXA). DXA measurements were made using half-body scans^(^^17^^)^. The same DXA scanner (Lunar Prodigy, Encore software version 12.3) was used throughout the study to avoid variations in the analysis of the scan results. However, due to weight limitations, three subjects were scanned in a DXA scanner with larger table size and weight capacity (Lunar iDXA, Encore software version 12.3).

### Biochemical measures

Blood samples were collected after an overnight fast (10–12 h) and were analysed for glucose, insulin, C-peptide, TAG, total cholesterol, HDL, LDL and high-sensitive C-reactive protein. Preparation and analysis of the blood samples have been described in detail elsewhere^(^^16^^)^. Within- and between-assay CV were below 3·5 and 6·5 %, respectively, for all biochemical measures.

### Statistical analysis

All statistical analyses were made in R version 2.14.0 (www.r-project.org).

Descriptive data summaries (age, body weight, BMI) are presented as mean values and standard deviations. The efficacy of the LCD on all endpoints was modelled in a mixed linear model with week as fixed effect and subject as random effect. All analyses were based on intention-to-treat with baseline observations carried forward for missing data (*n* 2), except for body weight where the last observation (measured at the weekly meeting with the dietitian) was carried forward for missing data. Subanalyses were modelled in a mixed linear model including a week × group interaction and with subject as random effect. Data are presented as mean values with their standard errors.

*P* values correspond to likelihood ratio tests and *P* values <0·05 were considered significant. Figures and parameters were based on raw data, whereas conclusions (*P* values) were based on model estimations.

Power calculations estimating the number of subjects to be included were based on the primary endpoint of the main study^(^^16^^)^. However, a sample size calculation, based on data from a previous study using a 3347–4184 kJ (800–1000 kcal) LCD for 8 weeks^(^^18^^)^, showed that with our sample size we were powered to detect an 8 % weight loss in obese subjects (corresponding to a mean weight loss of 11·1 kg, an sd of 3·3 kg and a minimal relevant difference of 8 kg (8 % weight loss); α = 0·05, β = 0·8).

## Results

### Descriptive data

In all, eight males and twenty-two females were included in the study. Average age was 38·8 (sd 10·4) years, with a mean body weight at baseline of 135·1 (sd 19·2) kg and BMI of 46·0 (sd 4·4) kg/m^2^. Two subjects dropped out before the first follow-up visit (week 7), due to personal problems and an inability to comply with the LCD.

### Weight loss

**Fig. 2.**
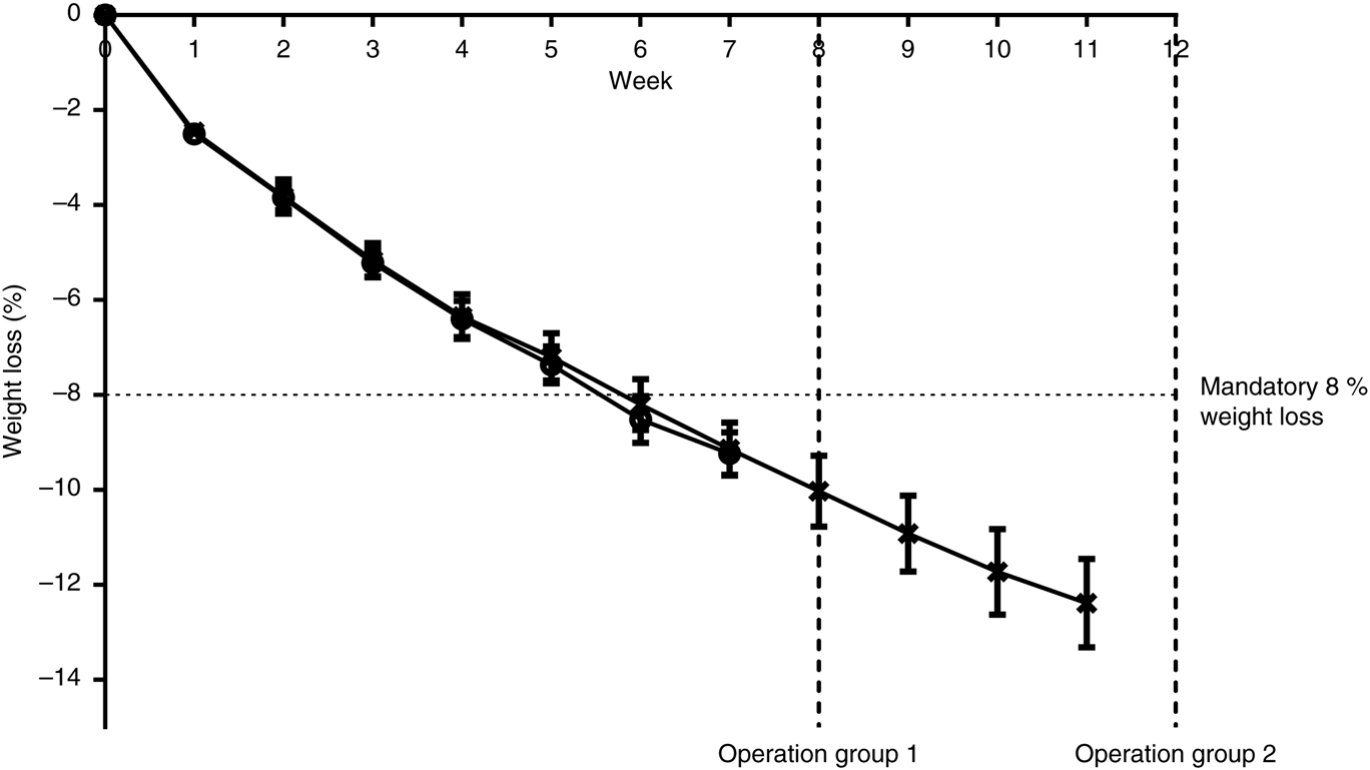
Weekly weight loss (%) measured from week 0 to 7 (*n* 30; groups 1 and 2; ○) and from week 0 to 11 in group 2 only (*n* 15; X). Group 1 were operated in week 8; group 2 was operated in week 12. Values are means, with standard errors represented by vertical bars.

The mean weight loss at week 7 was 9·3 (sem 0·5) %, corresponding to a weight loss of 12·7 (sem 0·8) kg (Fig. 2).

Of the included patients, 77 % reached their preoperative target weight, and this was on average achieved at week 5·4 (sem 0·3). Weight loss in the group that reached their target weight before week 7 (*n* 23) was 10·2 (sem 0·3) %, corresponding to a weight reduction of 14·1 (sem 0·7) kg, whereas mean weight loss for the remaining subjects in week 7 (*n* 7) was 6·1 (sem 0·9) %, corresponding to a weight reduction of 7·9 (sem 1·2) kg. Percentage weight loss at week 7 did not predict percentage excess weight loss 18 months postoperatively (*r* −0·40; *P* = 0·07).

A further 3·9 (sem 0·5) % reduction in body weight was observed from week 7 to 11 in the fifteen patients scheduled for BS in week 12 (*P* < 0·01). All of the twenty-eight subjects who completed the follow-up visits reached their target weight prior to surgery in weeks 8 and 12, respectively. Mean dietary compliance was 4·3 (sem 0·1) from week 0 to 7, and remained unchanged until week 11 (4·1 (sem 0·2); *P* = 0·16). Compliance was positively associated with percentage weight loss from week 0 to 7 (*r* 0·59; *P* < 0·001).

FFM and fat mass comprised 41·6 and 58·4 % of the weight loss from week 0 to 7, respectively. A further reduction in both fat mass and FFM (5·0 (sem 0·8) and 2·3 (sem 0·7) %, respectively) was observed from week 7 to 11 (both *P* < 0·01).

**Fig. 3.**
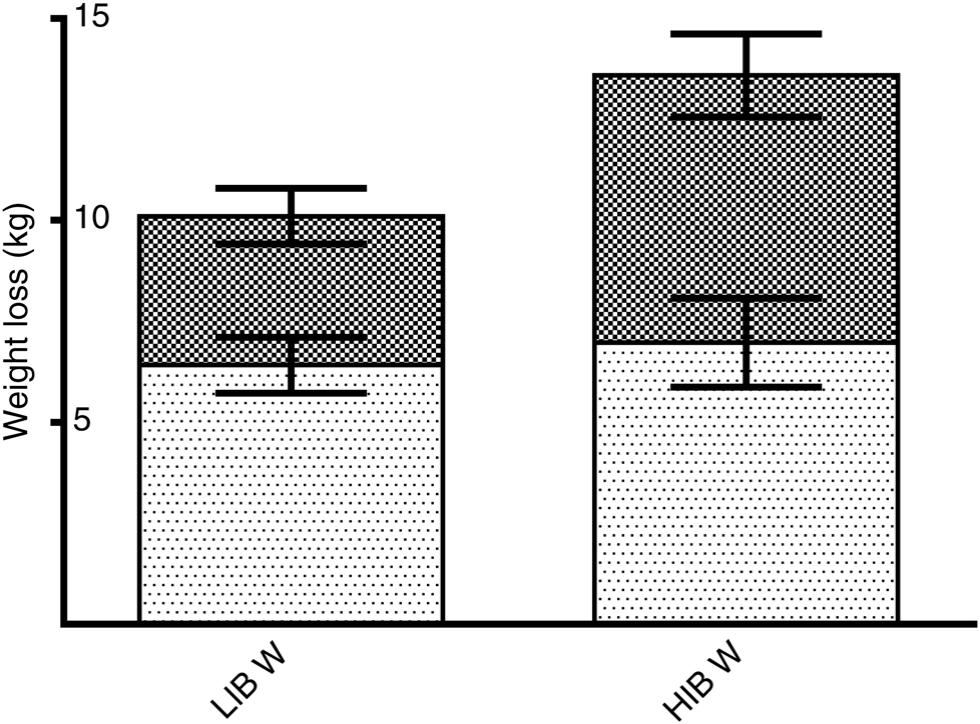
Fat-free mass (▒) and fat mass (░) loss in patients with higher initial body weight (HIBW; above the 50th percentile corresponding to body weight ≥135 kg) and patients with lower initial body weight (LIBW; below the 50th percentile corresponding to body weight <135 kg). Values are means, with standard errors represented by vertical bars. A mixed linear model was used to test for differences between the groups. There was a significant difference between the LIBW and HIBW groups.

A *post hoc* analysis comparing patients with higher initial body weight (HIBW) (above the 50th percentile, corresponding to body weight ≥135 kg) and patients with lower initial body weight (LIBW) (below the 50th percentile, corresponding to body weight <135 kg) was carried out. This analysis revealed that the HIBW group experienced a greater relative weight loss (9·8 (sem 0·5) *v*. 8·7 (sem 0·7) %; *P* < 0·01) and that FFM accounted for a larger part of the weight loss compared with patients in the LIBW group (46 *v*. 36 %, respectively, *P* = 0·02, corresponding to 6·3 (sem 1·0) and 3·5 (sem 0·7) kg, respectively, Fig. 3).

### Blood parameters

From week 0 to 7 fasting glucose, insulin and C-peptide decreased by 8·2 (sem 1·8), 28·6 (sem 6·4) and 15·4 (sem 4·5) %, respectively (all *P* < 0·01). TAG, total cholesterol, HDL and LDL decreased by 9·7 (sem 4·7), 21·7 (sem 2·0), 23·5 (sem 2·5) and 23·1 (sem 2·2) %, respectively (Table 1; all *P* < 0·05). No further decrease in any of these parameters was observed from week 7 to 11.

**Table 1.** Changes in anthropometric and biochemical parameters after a 7-week low-energy formula diet treatment in thirty obese patients (Mean values with their standard errors)

	Week 0 (*n* 30)		Week 7 (*n* 30)		Change week 0–7	
Outcome	Mean	sem	Mean	sem	Mean	sem
Body weight (kg)	135·1	3·5	122·4	3·0	−12·7**	0·8
BMI (kg/m^2^)	46·0	0·8	41·8	0·7	−4·2**	0·2
Hip circumference (cm)	143·4	2·3	134·2	2·2	−9·2**	1·3
Waist circumference (cm)	129·9	2·5	119·8	2·4	−10·1**	0·9
FFM (kg)	66·2	2·0	61·3	1·7	−4·9**	0·6
FM (kg)	67·4	2·2	60·6	2·0	−6·9**	0·7
Glucose (mmol/l)	5·9	0·2	5·4	0·1	−0·5**	0·1
Insulin (pmol/l)	131·9	16·5	80·5	7·2	−51·4**	13·9
C-peptide (pmol/l)	1192·6	84·5	934·2	36·9	−258·4**	76·6
hs-CRP (mg/l)	11·8	1·5	8·5	2·0	−3·3**	1·1
TAG (mmol/l)	1·6	0·2	1·3	0·1	−0·3*	0·1
Total cholesterol (mmol/l)	5·4	0·2	4·2	0·1	−1·2**	0·1
HDL (mmol/l)	1·2	0·1	0·9	0·1	−0·3**	0·1
LDL (mmol/l)	3·4	0·1	2·6	0·1	−0·8**	0·1
Systolic BP (mmHg)	126·3	2·7	119·1	2·0	−7·1**	2·3
Diastolic BP (mmHg)	87·4	2·0	80·1	1·7	−7·3**	1·8
HR (beats/min)	67·8	1·7	62·9	1·3	−4·9**	1·3

FFM, fat-free mass; FM, fat mass; hs-CRP, high-sensitive C-reactive protein; BP, blood pressure; HR, heart rate.

Mean value of change from week 0 to 7 was significant: *P < 0·05, **P < 0·01.

### Heart rate and blood pressure

Heart rate, systolic and diastolic blood pressure decreased by 4·9 (sem 1·3) beats/min, 7·1 (sem 2·3) and 7·3 (sem 1·8) mmHg, respectively (all *P* < 0·01), with no further decrease observed from week 7 to 11. At baseline, six patients had blood pressure above the normal range (>140/90 mmHg), and only one of these patients was pharmacologically treated for this. Blood pressure normalised in five of these six patients at week 7. An additional five patients were on antihypertensive medication with blood pressure within the normal range at baseline. Medication was withdrawn in one of these patients at week 7.

### Side effects

The most commonly reported side effects were headache, fatigue, constipation, dizziness and upper respiratory infections (reported by 57, 50, 43, 33 and 30 %, respectively). Other less-reported side effects included cold intolerance, hunger, abdominal pain, irritability, dry skin, diarrhoea, flatulence and bad breath. No severe adverse events were reported.

## Discussion

In the present study, we demonstrated that for the vast majority of patients, it is possible to rapidly achieve an 8 % preoperative weight loss by implementation of a 7-week LCD in combination with dietary counselling. The mean weight loss of 9·3 (sem 0·5) % is comparable with that found by Papadaki *et al.* reporting a weight loss of 11·1 % in overweight and obese adults who followed an LCD for 8·2 weeks^(^^18^^)^. Our patients generally reported good compliance with the LCD during the 7 weeks. Although compliance relies on self-evaluated measures with inherent limitations in terms of validity, we found that reported compliance explained 35 % of the variation in weight loss at week 7. With a dropout rate of only 7 %, an LCD management approach seems to be a viable option for severely obese patients to obtain preoperative target weight. Compliance with LCD has been shown to decrease over time^(^^2^^)^; however, reported compliance was unchanged from week 7 to 11 and weight loss continued, indicating that an LCD programme for 11 weeks is still reasonable from a compliance perspective. The baseline characteristics and reported compliance of the seven subjects who failed to achieve preoperative target weight at week 7 were not different from the rest of the study group (data not shown).

Although loss of FFM is unavoidable in a weight-loss process based on dietary changes in patients with obesity^(^^19^^)^, a relative preservation of FFM is beneficial due to its higher metabolic activity compared with fat^(^^19^^,^^20^^)^. It has been suggested that the acceptable amount of FFM loss in obese subjects (mean BMI = 35 kg/m^2^) is within the range of 22–30 % of total weight loss^(^^21^^)^, and studies evaluating the effect of energy-deficient diets (2092–5021 kJ (500–1200 kcal)) in obese subjects typically report loss of FFM to account for 19–36 % of total weight loss^(^^18^^,^^22^^–^^26^^)^. Thus, the weight loss induced by the LCD in the present study resulted in a considerable reduction of FFM (41·6 % of total weight loss). The reduction in FFM is high, especially considering the relatively high protein content (39 E%) of the LCD. However, it is questionable whether the LCD can be defined as a high-protein LCD, when applied in severely obese patients. A daily protein intake of 0·83 g/kg per d is recommended in healthy adults in energy balance^(^^27^^)^, but there are no formal recommendations for protein requirement during weight loss in the severely obese. As the mean protein intake in the present study was 0·75 (sem 0·10) g/kg per d at baseline, it can be argued that the protein intake during the LCD cannot be considered high in this population. In addition, the large negative energy balance in these severely obese patients might be an underlying cause of the excessive loss of FFM. In a systematic review, Chaston *et al.*^(^^19^^)^ reported that the loss of FFM following dietary interventions increased with the degree of energy deficit and consequently the rate of weight loss. This is supported by our subanalysis, which revealed that patients with HIBW experienced a greater loss of FFM compared with patients with LIBW (46 *v*. 36 %). Comparing the protein intake/kg body weight per d at baseline in the HIBW and the LIBW, we found a lower intake in the HIBW (0·66 (sem 0·06) *v*. 0·84 (sem 0·05) g/kg per d; *P* < 0·01). These results indicate that a 4184 kJ (1000 kcal) LCD with a protein content of 39 E% is not sufficient for preservation of FFM in obese patients in negative energy balance, especially not in severely obese patients with an initial body weight >135 kg.

No severe adverse effects of the LCD were reported. The most commonly reported side effects were headache, fatigue, constipation and dizziness, which are well-known side effects following an LCD^(^^28^^–^^30^^)^. Levels of fasting glucose, insulin, C-peptide, cholesterol, TAG, HDL and LDL decreased from week 0 to 7, as previously demonstrated^(^^31^^,^^32^^)^. Interestingly, the period from week 7 to 11 did not induce any additional beneficial effects, suggesting that energy restriction has an immediate effect on these blood parameters. In agreement with previous findings^(^^28^^)^, the LCD was also associated with a reduction in systolic and diastolic blood pressure, and four out of five subjects falling into the hypertensive range at baseline were normotensive at week 7. We found no association between preoperative weight loss and weight loss 18 months postoperatively. The evidence is inconclusive on whether or not a relationship exists. Consistent with our findings, some studies report no association^(^^9^^,^^33^^,^^34^^)^, whereas others have found a beneficial effect of preoperative weight loss on postoperative weight loss^(^^7^^,^^10^^,^^11^^)^. Taken together, these results suggest that a 7-week preoperative LCD treatment optimises the health of patients prior to surgery, but has no effect on long-term weight outcome.

As nutritional counselling is an important aspect in the treatment of obesity^(^^35^^,^^36^^)^, a limitation of the present study is that the results may not be directly applicable to candidates for BS who do not receive weekly nutritional counselling and encouragement from an experienced health professional. Furthermore, the exclusion of diabetic patients and subjects who were unwilling to comply with the LCD could have affected our results, as this group may be less prone to weight loss^(^^37^^)^. Another limitation is the DXA, which has not been fully evaluated as an assessment tool for body composition in severely obese patients, and has been reported not to provide valid data under dynamic weight loss due to inaccurate assumptions of a constant FFM hydration^(^^38^^)^.

In conclusion, our results suggest that it is possible to obtain preoperative weight loss of 8 % with an LCD within 7 weeks. The weight loss at week 7 was accompanied with an improved metabolic profile and a reduction in blood pressure, with no further improvements after an additional 3·9 % weight loss at week 11. The mechanisms behind the excessive reduction in FFM in highly obese subjects following LCD needs further investigation, including the potential for optimisation of LCD formulas used in order to limit loss of metabolically active tissue.
